# Assessment of water quality and health hazards using water quality index and human health risk evaluation in district Talagang Pakistan

**DOI:** 10.1038/s41598-025-89932-y

**Published:** 2025-02-12

**Authors:** Nazir ur Rehman, Mumtaz Ali Khan, Shuja Ullah, Afra Siab, Fakhrul Islam, Ahmed Elbeltagi, Ali Salem

**Affiliations:** 1 Department of Geology, Khushal Khan Khattak University, Karak, 27200 Khyber Pakhtunkhwa Pakistan; 2 Department of Earth & Environmental Sciences, Bahria University, Islamabad, Pakistan; 3 NCE in Geology, University of Peshawar, Peshawar, 25130 Khyber Pakhtunkhwa Pakistan; 4 Agricultural Engineering Dept, Faculty of Agriculture, Mansoura University, Mansoura, 35516 Egypt; 5 Civil Engineering Department, Faculty of Engineering, Minia University, Minia, 61111 Egypt; 6 Structural Diagnostics and Analysis Research Group, Faculty of Engineering and Information Technology, University of Pécs, Pécs, 7624 Hungary

**Keywords:** Surface & subsurface water, Water quality index, Health risk assessment, Irrigation water quality, Environmental sciences, Geochemistry

## Abstract

This work was carried out for the determination of the water quality in the Talagang District of Pakistan, as water is essential for agriculture and drinking uses. This study aims to assess the water quality for irrigation, drinking, and health risks using the Water Quality Index (WQI) and Human Health Risk Assessment (HHRA) tools to identify regions with contaminated water, and to evaluate the associated risks. A total of 98 water samples were taken at various points from diverse sources such as hand pumps, streams, springs, dug wells, and tube wells for physio-chemical assessment. In the current study, the effectiveness of the irrigation water quality index (IWQI), human health risk assessment (HHRA), and water quality index (WQI) tools have been assessed. The characteristics of subterranean water are influenced by evaporation, ion exchange, rock-water interaction, and parent-rock weathering, as shown by the Piper and Gibbs diagram. According to the WQI results, the water quality is 20. 89% and 27.46% of the sample sites are moderate and poor, making them unfit for human intake. Based on HHRA, compared to adult males and females in the study area, children are deemed to be at a higher risk. A larger number of the sample localities are appropriate for irrigation purposes. The study assists in identifying contaminated regions and in monitoring newly implemented remediation actions to manage the source of contaminants in the study area.

## Introduction

Surface and subterranean water are essential sources of drinking, farming, industrial, and domestic uses worldwide and also have a substantial effect on shaping the quality of lives and sustainability of societies^1^. Due to rapid growth and population increase, natural and human actions such as industry, urbanization, mining, and agriculture have resulted in water depletion and impairment issues^2,3^. Water quality degradation and depletion have emerged as significant global challenges, directly impacting public health, agriculture, and the environment^4,5^. The poor quality of water poses both direct and indirect health risks to the communities that rely on it, often leading to substantial public health issues and increased costs for water treatment and rehabilitation^6^. Direct health risks are associated with the consumption of contaminated water, such as heavy metal contamination, which can cause serious illnesses^7,8^. Indirect health risks occur when contaminated water is used for irrigation, affecting agricultural crops, horticulture, and aquaculture, leading to bioaccumulation of toxins in the food chain^9^.Heavy metals including Zn, Cu, and Mn are naturally occurring in water in trace amounts and are significantly essential for human metabolism and the growth of living things^10^. However, excessive amounts of these metals pose chronic and acute health issues. Other heavy metals including Pb, Cd, As, Cr, and Ni are severely toxic although in very low concentrations^11^. For example, the higher concentration of Pb is known to harm the development of the brain in children. Exposure to elevated concentrations of Cd causes chronic and acute diseases such as skeletal and kidney damage. The As causes many health problems in humans such as skin lesions, and cancer of the liver, brain, stomach, and kidney^12,13^. Higher intakes of Cr and Ni have been linked with liver, kidney, and heart problems^14^. The WQI is a handy means for evaluating the quality of water that is appropriate for residential practice. The weighted arithmetic and integrated WQI are extensively used in India for assessing surface and subterranean water because it yield results with greater accuracy^15^^16^. investigatedthe chemistry and quality index of groundwater in northwest China and noticed that 11.43% of sample locations had poor water quality, and 17.14% had very poor water quality. Similarly^17^, used weighted overlay analysis to assess groundwater quality for drinking and irrigation purposes in Bangladesh, revealing that 90% of water from deep wells and 57.6% from shallow wells were suitable for human consumption, according to the Drinking Water Quality Index (DWQI).

Several recent studies have employed various techniques to assess water quality, including the use of WQI, which integrates multiple physicochemical variables into a single dimensionless value representing overall water quality^18,19,20,21,22^. The WQI is an assessment model that can be used for integrating a variety of physicochemical variables into a dimensionless value that may depict the overall quality of the water^18,20^. n Pakistan, water quality contamination has been reported in several regions, affecting both surface and groundwater resources^11^. Given the importance of water for human health, agriculture, and overall well-being, it is crucial to evaluate the water quality in various regions. The primary objective of this study is to assess the surface and subsurface water quality for irrigation, drinking, and health risks in Talagang District, Pakistan, using the Water Quality Index (WQI) and Human Health Risk Assessment (HHRA) tools. This research aims to evaluate the hydro-chemical parameters of groundwater in the study area for both irrigation and drinking purposes, and to assess the associated health risks using the WQI and HHRA models. The findings will contribute to identifying areas where water quality poses health risks and help in formulating strategies for water management and remediation.

## Materials and methods

### Study area

**Fig. 1:**
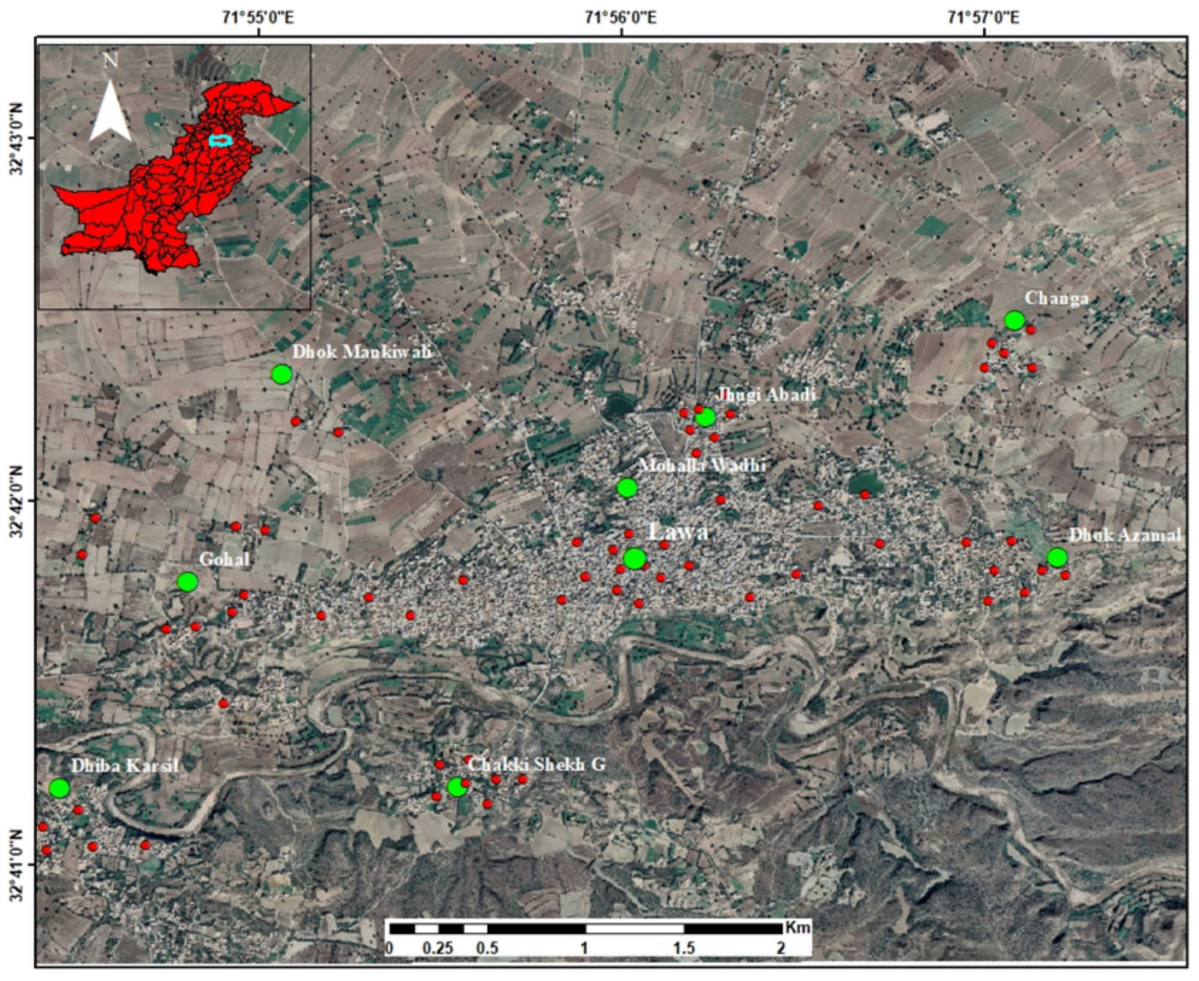
Location map of the study area exhibiting sampling sites (ArcGIS Desktop (ArcMap) 10.8.2).

**Fig. 2:**
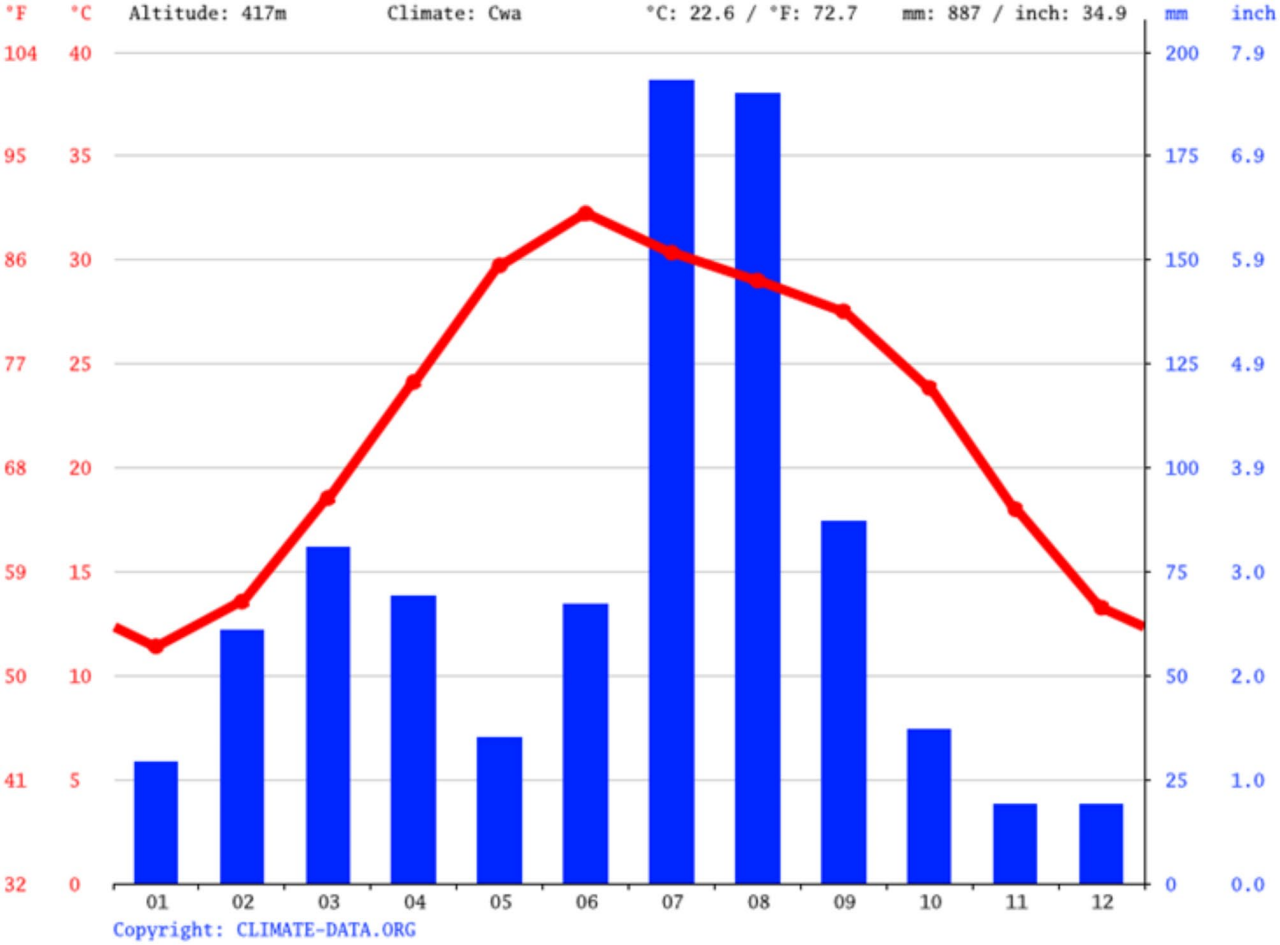
Climate graph/Weather by month.

The study area is located to the southwest of Talagang District in the Punjab province of Pakistan. It lies at 508.0 m above sea level with a geographical location of 32° 41′ 47.07″ N, 71° 55′ 56.21″ E, covering 977.7 km^2^ area (Fig. 1). In the Lāwa area, the climate is hot and moderate. June is the warmest month with a mean temperature of 29.4 °C/84.92 °F and January has the lowest average temperature of the year i.e. 11.4 °C/52.5 °F. The mean temperature in Lāwa is 222.6 °C/72.27 °F. In the winter, there is much less rainfall than in the summer. The rainfall here is about 8887 mm/34.98 inches per year. The month characterized by the lowest precipitation levels is November, exhibiting a mere 19 mm/0.7 inch of rainfall. The highest amount of precipitation occurs during July, with an average quantity reaching up to 193 mm/7.6 inches (Fig. 2). The residents of the study region rely heavily on subterranean water sources for domestic, drinking and irrigation purposes.

Geologically, the study region is made up of sedimentary rocks with ages ranging from the Miocene to Pleistocene periods, including conglomerates, sandstone, siltstone, shale, etc. Mostly these rocks are molasses.

The Molasse sediments consist predominantly of sandstone, shale, claystone, and siltstone, which are key lithological units influencing groundwater chemistry^23^. These formations are part of the Tertiary molasse deposits, commonly observed in sedimentary basins and foreland regions of the Himalayan orogeny^24^.

Sandstone layers within the Moasse sediments serve as primary aquifers due to their high porosity and permeability, facilitating groundwater storage and flow. However, these layers also act as conduits for the migration of contaminants from surface activities, such as agricultural runoff and industrial effluents^25^. Shale and claystone, on the other hand, possess low permeability, acting as confining layers that may restrict vertical movement of water and contaminants but can also promote localized accumulation of solutes, altering water chemistry^26^.

Additionally, the mineralogical composition of these sediments, including feldspar, quartz, and clay minerals, contributes to geochemical processes like ion exchange, dissolution, and precipitation, impacting the groundwater’s quality. For example, clay minerals in shale and claystone can release cations such as calcium, magnesium, and potassium into the water through weathering processes^25^. Furthermore, the presence of sulfides or carbonate minerals within these sediments may lead to acidification or alkalinization of groundwater, depending on the hydrogeological conditions^26^.Siltstone layers, while less permeable than sandstone, provide intermediate pathways for groundwater flow, potentially facilitating the transport of fine particulate matter and dissolved contaminants. Overall, the heterogeneity in the lithology of the Moasse sediments creates a complex hydrogeological environment that governs groundwater chemistry and the distribution of potential contaminants^23,24^. The bulk of groundwater is drawn from the upper and middle terraces’ unconsolidated surficial deposits of silt, sand, and gravel; from active and inactive floodplains; and from piedmont deposits resulting from the deposition of coarse-grained material from the highlands^27^.

### Sampling and analysis

Water samples were haphazardly taken from TW, DW, HP, springs, and streams that are present in the research area at 98 different sample sites (Fig. 1). Groundwater samples such as TW, DW, and HP were taken after flushing for 15–20 min in 250 mL and 500 mL in prewashed glass and polyethylene bottles: out of which 3250 mL is acidified with nitric acid (HNO_3_). Physical characteristics of water, such as pH, EC, and TDS, are measured at the sample’s site with a Multi-Parameter Aquaprobe AP-7000, UK. Prominent anions and cations, including Ca, Mg^2+^, Na^2+^, K^2+^, HCO_2_^-^, CO_2_^2-^, Cl^–^, SO_4_^2-^, NO_2_^–^, F^–^ were examined using conventional techniques as advised by the APHA (2005). Using an Atomic Absorption Spectrophotometer (AAS, AAnalyst 700, PerkinElmer), major ions like Ca^2+^, Mg^2+^, Na^+^, K^+^, and heavy metals such as Fe, Mo, Cr, Ni, Cu, and Zn were examined. The precision of analytical results for the main anions and cations is expressed in milliequivalents per liter (imeq/L), using the charge balance error (CBE) equation. The permissible CBE threshold is approximately ± 10%.

1 $$\:CBE=\sum\:\text{C}\text{a}\text{t}\text{i}\text{o}\text{n}\text{s}-\sum\:\text{A}\text{n}\text{i}\text{o}\text{n}\text{s}\:\sum\:\text{C}\text{a}\text{t}\text{i}\text{o}\text{n}\text{s}+\sum\:\text{A}\text{n}\text{i}\text{o}\text{n}\text{s}\times\:100$$

### Hadrochemical facies and mechanisms governing surface and subterranean water chemistry

Using a Piper trilinear plot^28^ is the most effective way to display the chemical composition of the water sample. It has two triangles that stand in for cations and anions, respectively and a diamond that provides more detail on the anions and cations’ summaries. The hydro-chemical facies of both surface and groundwater are contingent upon the characteristics of earth metals, specifically weak and alkaline acids. It assists in determining the lithology and flow pattern component of the research area. Gibbs diagram is used to find key variables which have a significant impact on how groundwater quality changes. It suggested a graphical depiction of the process by which water quality can be altered, and it highlighted three main structures that control the hydro-chemical facies of water. These are (1) evaporation, (2) rock-water interaction, and (3) precipitation dominance influencing the water’s chemical composition.

### Water quality index (WQI)

The WQI is an effective tool for displaying the overall effect of numerous water quality indicators as a single number which can be attributed to a particular quality class. In the current study, every factor has a distinct weight based on its significance and effect on the quality of the water suitable for drinking purposes^29,30^. For the WQI calculation, about nine parameters were selected; nitrate was given a high weight of five, while Ca and Mg were given a low weight. The following equations were used for the WQI calculations^31,20,1^:

2 $$\:{W}_{i}=\frac{{W}_{i}}{\sum\:_{i=1}^{n}{W}_{i}}$$

3 $$\:{q}_{i}=\frac{{C}_{i}}{{S}_{i}}\times\:100$$

4 $$Sl = W_{i}\times {q}_{i}$$

5 $$ WQI = \sum\limits_{{i = 1}}^{n} {SI}   $$

Where Wi is the relative weight, wij is the given weight for each variable, qi is the quality rating, Ci is the concentration of a given variable, Si is the standard value, and SI is the subindex value. According to the WQI values, water in the research region has been further categorized as excellent (WQI < 50), good (50*-100), poor (100–200), very poor (200i-300), and inappropriate for ingesting (WQI > 300).

### Irrigation water quality indices (IWQI)

It is crucial to assess groundwater for agricultural purposes because contaminated irrigation water has a negative effect on human health, plant growth, soil quality, and crop productivity^32^. People in the study region mostly rely on agriculture for their basic needs. Assessing the quality of water for irrigation is becoming more and more important. The irrigation indices including sodium adsorption ratio (SAR), residual sodium carbonate (RSC), permeability index (PI), magnesium hazard (MH), percentage sodium (%Na), potential salinity (PS), residual sodium bicarbonate (RBSC) and kellly ratio (KR) are computed by utilizing Eqs. (6–13).

Sodium absorption ratio (SAR):

6 $$\:\varvec{S}\varvec{A}\varvec{R}=\:\frac{\varvec{N}\varvec{a}}{\sqrt{\frac{\varvec{C}\varvec{a}+\varvec{M}\varvec{g}}{2}}}$$

Residual sodium carbonate (RSC):

7 $$\:RSA=\left(C{O}_{3}+HC{O}_{3}\right)+\left(Ca+Mg\right)$$

Percentage Sodium (%Na):

8 $$\:\mathbf{\%}\varvec{N}\varvec{a}=\frac{\varvec{N}\varvec{a}+\varvec{K}}{\varvec{N}\varvec{a}+\varvec{K}+\varvec{C}\varvec{a}+\varvec{M}\varvec{g}}\times\:100$$

Magnesium hazards (MH):

9 $$\:MH=\:\frac{Mg}{Ca+Mg}\times\:100$$

Permeability Index (PI):

10 $$\:\varvec{P}\varvec{I}=\frac{(\varvec{N}\varvec{a}+\varvec{H}\varvec{C}{\varvec{O}}_{3})}{\varvec{C}\varvec{a}+\varvec{M}\varvec{g}+\varvec{N}\varvec{a}}$$

Potential salinity (PS):

11 $$\:PS=\:\frac{Cl+1}{2SO4}$$

Residual Sodium Bicarbonate (RBSC):

12 $$\:\varvec{R}\varvec{B}\varvec{S}\varvec{C}=\varvec{H}\varvec{C}{\varvec{O}}_{3}-\varvec{C}\varvec{a}$$

Kelly ratio (KR)

13 $$\:KR=\frac{Na}{(Ca+Mg)}$$

### Human health risk assessment (HHRA)

The HHRA is an assessment technique that shows the effects of drinking extremely polluted water on human health. The findings specify the extent of adverse effects on human health in different environmental circumstances^33^. Different pollutants can get into the human body in a number of pathways, however drinking water is the most significant^34,35^. The effects can be classified into carcinogenic and non-carcinogenic risks. For that purpose, the chronic daily intake (CDI) and hazard quotient (HQ) values were calculated for adults (> 187 years) and children (< 187 years) based on exposition and chronic risks using equations listed below^36^:

14 $$\:\varvec{C}\varvec{D}\varvec{I}=\varvec{C}\times\:\varvec{D}\varvec{I}/\varvec{B}\varvec{W}$$

Where C is the concentration of heavy metal in water expressed in µg/L, DI is the mean daily consumption found to be 0.2 L/d, and BW is the body weight of an individual calculated as 792 kg (for adults) and 630.68 kg (for children).

15 $$\:\varvec{H}\varvec{Q}=\frac{\varvec{C}\varvec{D}\varvec{I}}{\varvec{R}\varvec{f}\varvec{D}}$$

The oral toxicity reference doses (RffD) of heavy metals were considered^37^.

### Multivariate statistics

Pearson correlation matrix analysis has been conducted on the data to evaluate the statistical relationship between different water quality parameters. R studio, an open-source program, was used to conduct principal component analysis and hierarchical cluster evaluations. Kriging was utilized to map the irrigation water quality parameters and WQI’s spatial distribution^38,39^.

## Result and discussion

### Hydrochemistry classification

**Fig. 3:**
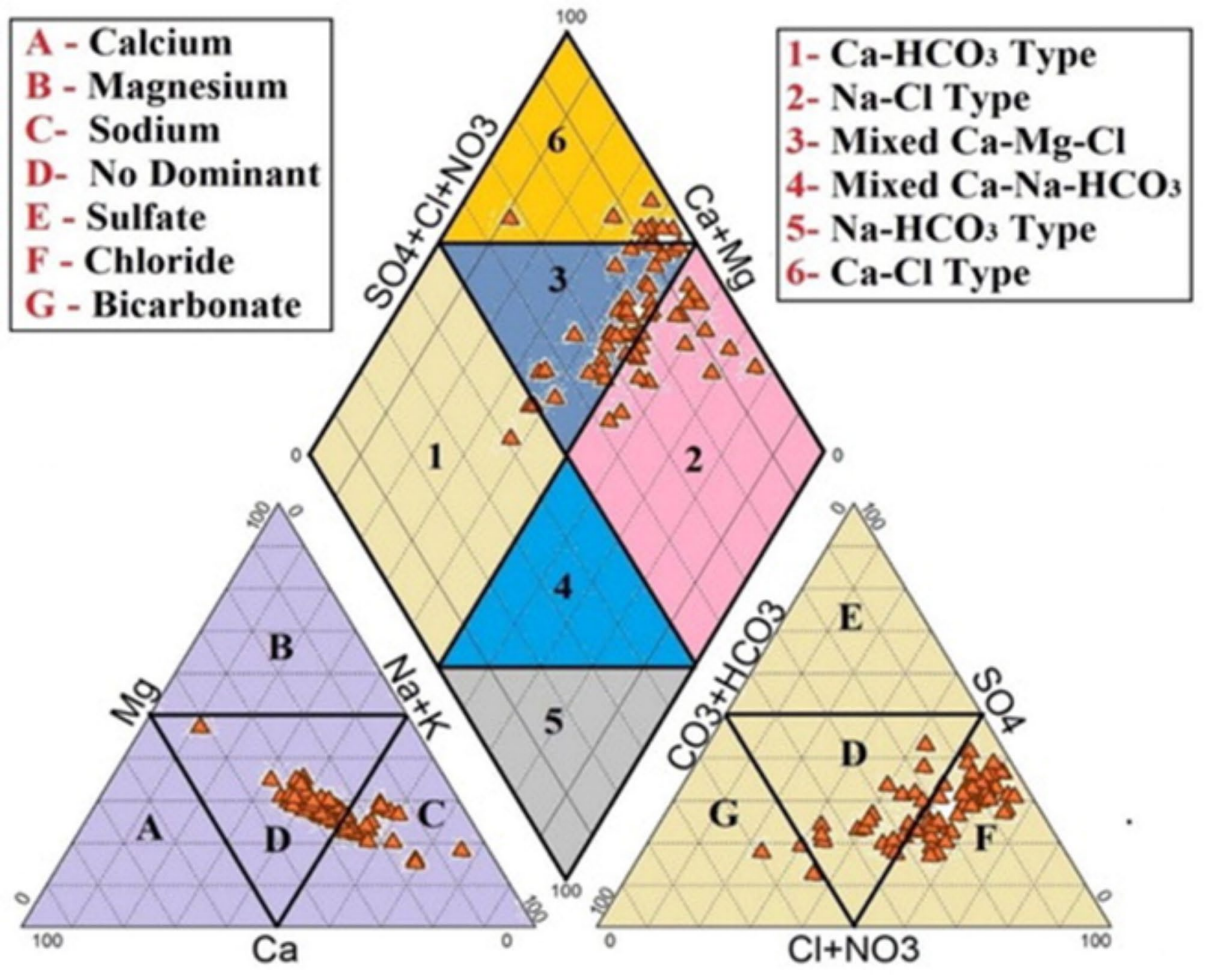
Piper-Hill diagram showing the hydrochemical facies in water sources of the research region.

The Piper plot indicates that the most prominent ions in the research region are the Ca ≫ Mg > Na of cations and NO_3_ ≫ SO_4_ > Cl of anion. Of the samples collected, 55.22% belong to the mixed Ca–Mg–Cl − type, 218.359% to the Ca–Cl − type, 911.914% to the Na–Cl type, and 94.417% to the Ca–HCO_3_ type of water (Fig. 3). Figure 3 depicts that Ca–Mg–Cl type ≫ Na–Cl type > Ca–Mg–HCO_3_ type. These two diagrams depict that rock–water interaction, base ion exchange processes, and aquifer nature are the significant variables affecting the quality of water in the research region.

### Gibbs Plot

**Fig. 4:**
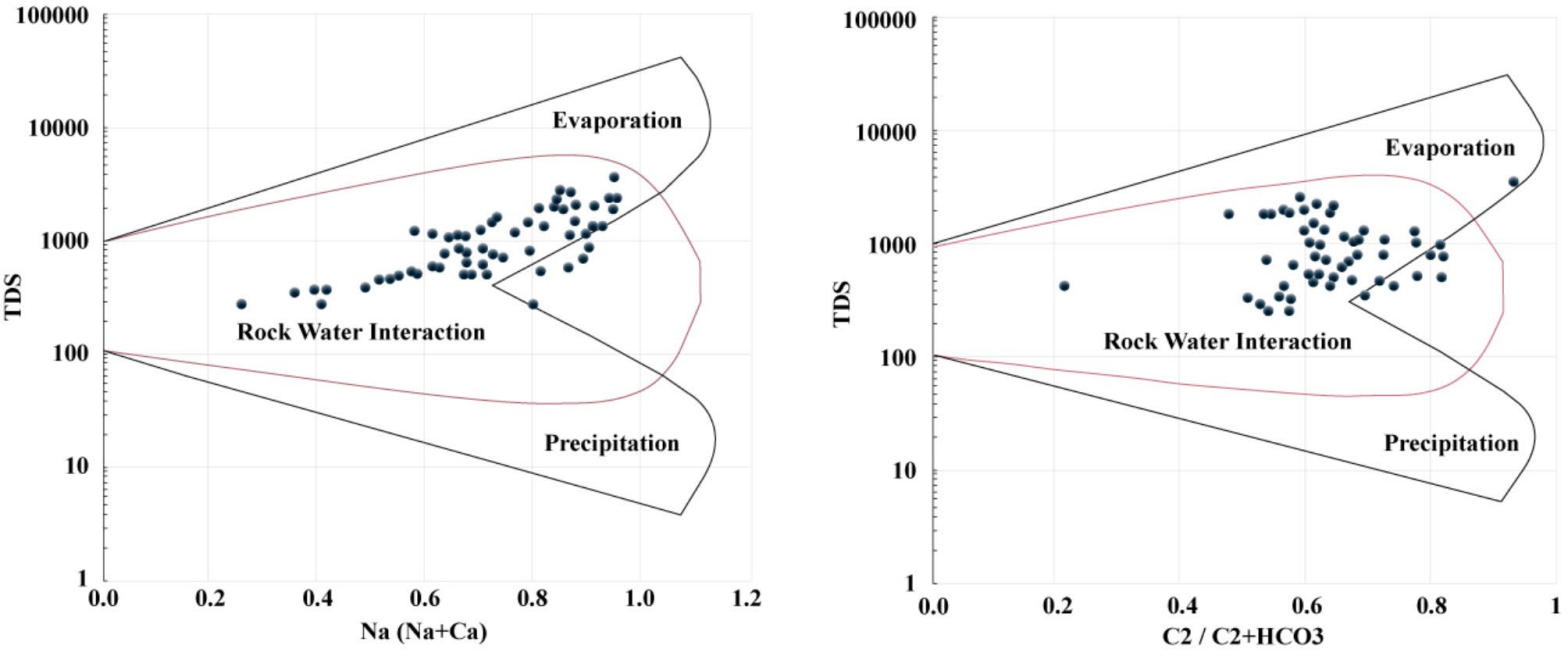
Mechanisms governing surface and subsurface water chemistry in the research region.

A Gibbs^40^ plot of surface and subsurface water samples in the research region is displayed in Fig. 4. It shows that a portion of the samples are found in the categories related to rock-water interaction in both the anion and cation plots. It was suggested that the ratio of Ca to Na and the amount of Na could rise as a result of salts leaching from rocks. The transition zone across evaporation and the dominant field of rock-water interaction in the research region is caused by the characteristics of aquifers and insufficient rainfall.

### Hydro-chemical characterization of groundwater

**Fig. 5:**
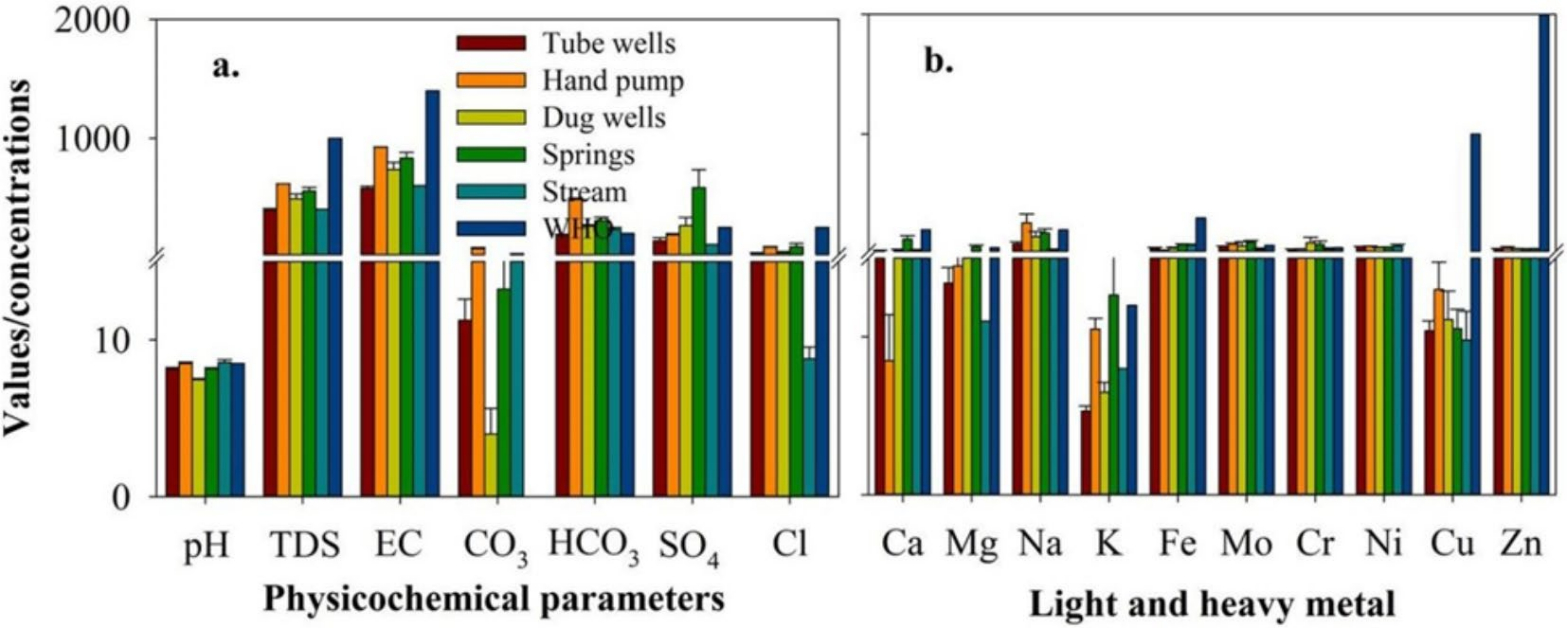
Water quality characteristics in the study area (**a**) physicochemical parameters, pH is unit less, EC is measured in µS/cm, TDS, CO_3_, HCO_3_, SO_4_, and Cl in mg/L, (**b**) light and heavy metals, Na, K, Ca, and Mg measured in mg/L; and Fe, Mo, Cr, Ni, Cu, and Zn in µg/L.

The statistical assessment of both surface and subterranean water of the research region is displayed in Table 1. The pH has a mean of 8.29 and a range of 7.46 to 9.05. Stream water exhibited the highest mean pH value (8.56), then the HP and DW 77.47 had the lowest (Fig. 5a; Table 1). The pH ranges from almost neutral to slightly alkaline. The disinfection of water is highly impacted by the pH of the water and a pH < 8 is ideal for efficient chlorination. However, an optimum pH value of 6.5-9.5 has been suggested for water supply networks to prevent deterioration. Moreover, a normal pH range of 6.5-8.4 is suggested for irrigation^41^ and 6.5-8.5 for drinking water purposes^42^. A primary cause of the elevated pH in the research region is the existence of calcareous and Charnockite nature aquifers^43^. Total dissolved salts in water (TDS) are a measurement that includes both organic and inorganic salts. The greatest mean values of TDS (6916 mg/L), EC (925 µS/cm), CO_3_ (72.5 mg/L), and HCO_3_ (4815 mg/L) were observed in samples collected from the HP and that of SO_4_ (584 mg/L) and Cl (87.4 mg/L) in spring water (Fig. 5a). These values were noticed in the drinking water standards define by^42^, excluding those of CO_3_, HCO_3,_ and SO_4_. The TDS and EC are significant factors when considering the utility of water for irrigation. Plants that are sensitive to salt may be affected by a TDS level higher than 9500 mg/L. For irrigation waters, it is ideal to have a TDS and EC value less than 5100 mg/L and 7150 µS/cm, respectively. According to TDS and EC levels, water from all the sources could be categorized as having a medium to high salinity class, which could be risky for plants that are not tolerant of high salinity^44^. Total Hardness (TH) represents the quantity of Ca and Mg ions in the water. TH does not have any health-based guideline value owing to the lack of conclusive evidence of it causing health problems, however, the concentration of TH may cause concerns for water portability and palatability. In general, water can be classified into four classes based on TH values, namely soft, defined as 10–160 mg/L; moderately hard, defined as 61–1201 mg/L; hard, defined as 1121–1180 mg/L; and very hard, defined as more than 1180 mg/L. In summary, most of the samples in the research region were categorized as hard or very hard. Overall, spring water samples show higher TH values in comparison with other sources. The order in which the major anion is present is Ca cation is in the order of Cl_43_.

**Fig. 6:**
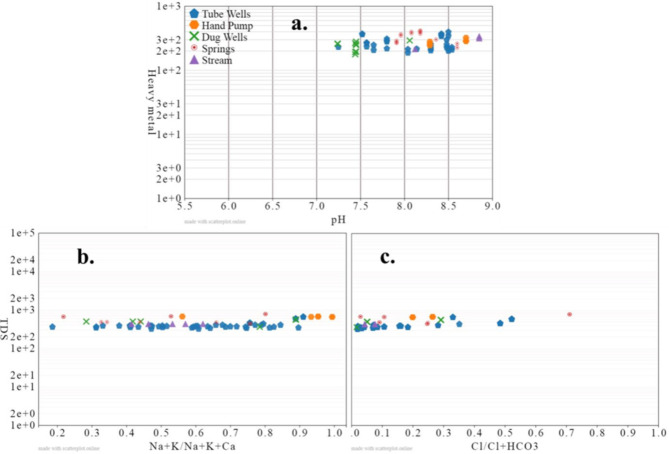
Water quality parameters on the (**a**) Ficklin plot (**b**) and (**c**) Gibbs plot in the research area, pH is units less, heavy metal in µg/L and other parameters in mg/L.

Among cations, the highest mean concentration (2527 mg/L) was found for Na in HP water and the lowest (5.31 mg/L) for K in Tube wells water (Fig. 5b; Table 1). Na, K, Ca, and Mg contents were observed within the acceptable boundaries of^42^, excluding the springs which show a greater total of Mg and K, and HP water that shows a higher concentration of Na. A higher concentration of Na can lead to a number of health problems such as stomach problems, hypertension, headaches, nausea, stroke, and kidney damage^45^ and that of K are abnormal protein metabolism, ovarian cysts, cystitis, fast heartbeat, and diminished renal function^46^. In the same way, increased Ca and Mg levels in drinking water have been scientifically observed to cause cardiovascular complications^47^. Mo showed the greatest mean concentration 95.9 µg/L in spring water, Fe comes in second (76.3 µg/L), and Cu has the lowest mean concentration (9.80 µg/L) in stream water (Fig. 5b; Table 1). The rest of the heavy metal concentrations were observed in between the two extremes. Heavy metal concentrations were found to be within the^42^ drinking water guidelines, with the exception of Mo in spring and hand pump water, and Ni in all studied sources of water (Fig. 6).

**Table 1:** Descriptive statistics of physicochemical characteristics including heavy metal in water sources of the study area.

Parameter	pH	Eh	TDS	HCO_3_	SO_4_	Cl	Ca	Mg	Na	K	SIO_2_	Cr	Mo	Ni	Cu	*P*	Zn	Fe
Hand Pump *n* = 43																		
Mean	8.32	64.3	643.72	455.3488	201.32	99.83	21.72	23.25	352.25	2.17	31.23	0.04	0.05	0.04	0.01	8.79	0.03	0.02
SE	0.07	8.09	28.82	17.251	33.79	15.59	5.73	5.77	46.35	0.25	2.01	0.00	0.00	0.00	0.00	0.70	0.00	0.00
Min	7.48	-58	277	270	13	1	1	1	31	0.5	10	0.01	0.02	0.01	0	2	0.01	0
Max	9.25	190	980	840	757	363	234	211	1952	9	64	0.08	0.17	0.11	0.02	18	0.07	0.12
Spring *n* = 23																		
Mean	8.11	85.30	523.91	490.2174	98.65	37.17	52.65	25.78	205.21	1.86	26.91	0.03	0.05	0.05	0.01	10.6	0.03	0.03
SE	0.1004	8.46	33.96	31.08779	28.05	11.52	11.10	5.25	22.85	0.21	2.39	0.00	0.00	0.00	0.00	0.84	0.00	0.01
Min	7.35	-61	301	230	8	1	2	1	33	1	6	0.01	0.01	0.01	0	2	0.02	0
Max	9.09	140	921	850	653	272	271	99	544	4	51	0.08	0.13	0.19	0.02	19	0.06	0.1
Stream Water *n* = 12																		
Mean	8.65	67.8	620.33	361.6667	275.66	51.08	58.25	39.91	224.91	3	18.83	0.04	0.05	0.05	0.01	9.16	0.04	0.02
SE	0.09	6.13	39.422	45.19039	89.67	3.56	24.15	13.443	35.65	0.60	2.14	0.00	0.00	0.01	0.00	1.33	0.01	0.01
Min	8.15	37	497	200	47	33	7	3	110	1	8	0.01	0.01	0.01	0	5	0.03	0
Max	9.15	116	930	760	1192	72	311	172	520	8	32	0.08	0.13	0.19	0.02	18	0.09	0.1
Dug Well *n* = 07																		
Mean	7.99	81.7	497.71	492.8571	72.57	42.85	44.85	26.57	160.71	1.71	31.28	0.03	0.05	0.04	0.00	9.71	0.03	0.04
SE	0.12	19.4	36.712	48.87858	21.53	14.36	11.14	3.63	21.03	0.28	4.50	0.00	0.01	0.01	0.00	1.74	0.00	0.01
Min	7.68	2.00	406	270	2	1	12	12	95	1	16	0.01	0.02	0.02	0.01	3	0.03	0
Max	8.48	125	680	640	145	103	78	38	253	3	47	0.06	0.1	0.08	0.01	17	0.07	0.08
Tube Well *n* = 13																		
Mean	8.44	29.7	588.61	426.9231	149.07	62.3	10.38	9.84	233.76	1.92	33.76	0.03	0.06	0.04	0.01	10.46	0.04	0.02
SE	0.12	26.9	67.641	30.99596	32.00	16.11	2.95	4.96	24.09	0.26	4.66	0.00	0.01	0.01	0.00	0.88	0.00	0.01
Min	7.7	-198	228	260	1	1	1	1	100	1	7	0.01	0	0.01	0	6	0.03	0
Max	8.95	161	965	620	372	210	34	68	403	3	56	0.06	0.17	0.08	0.02	18	0.05	0.06

### Hydrochemistry

**Fig. 7:**
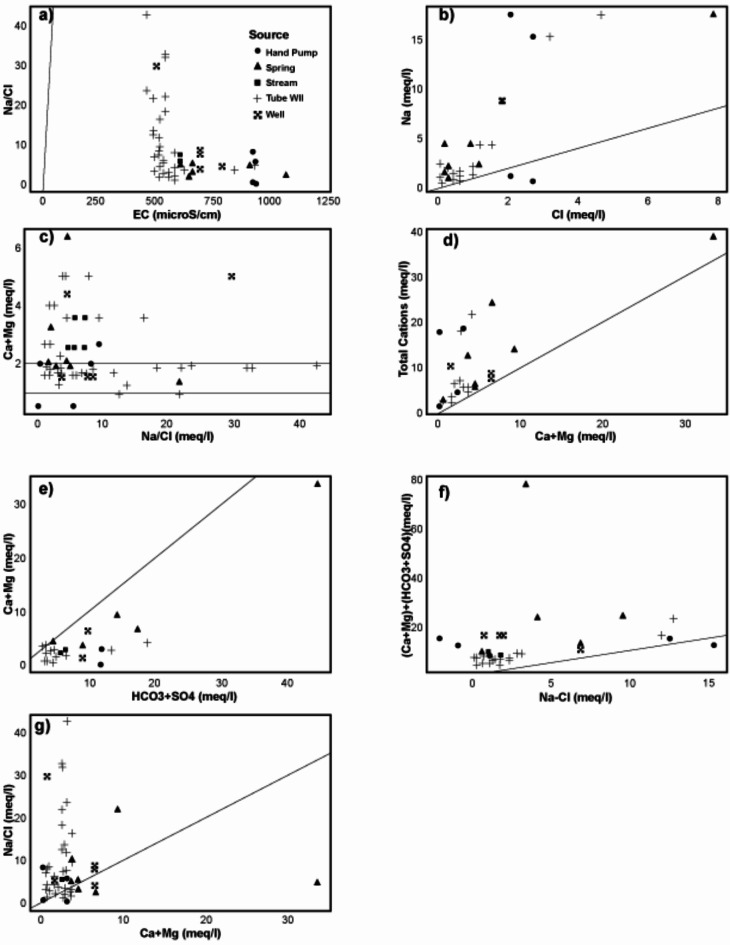
Bivariate plots with 1:1 equilines for (**a**) NaCl vs. EC (**b**) Na vs. Cl (**c**) Ca/Mg vs. Na/Cl (**d**) Total cations vs. Ca + Mg (**e**) Ca + Mg vs. HCO_3_ + SO_4_ (**f**) (Ca + Mg)+(HCO_3_ + SO_4_) and (**g**) Na/Cl vs. Ca + Mg. Values except EC are in meq/L.

The Ficklin plot model was employed to classify the water, based on pH and heavy metal concentrations^48^. The majority of the assessed water samples fell into the near-neutral and low heavy metal categories, according to the results (Supporting Information Fig. 6a). The Gibbs plot model helps divide water quality into three main groups based on where the hydrochemical components might come from: precipitation, rock weathering, and evaporation dominance. Plotting samples in the Gibbs model was done in the rock weathering dominance zone.(Supporting Information Fig. 6 b and c). These findings suggested that the area’s water chemistry is primarily shaped by rock-water interaction. Weathering of rocks may entail the disintegration of evaporite, carbonate, and silicate weathering. This could be further confirmed by the inverse relation between EC and Na: Cl plot shown in Fig. 7a, representing the insignificant contribution of evaporation from the water sources in the research region (Khangembam and Kshetrimayum, 2019). The ratio of Na to Cl is an important tool in discriminating the sources of chemical constituents of the water and the origin of salinity. In general Na/Cl ratio < 1 is attributed to halite dissolution and a value > 1 to silicate weathering^49,50^. The Na/Cl ratio in the research region lies in the range of 0.24–42.6 and is plotted within the Na field except for W-21 and W-22 (Fig. 7b). Similarly, the Ca/Mg ratio and its relation to the Na/Cl ratio could be used to understand the sources of hydrogeochemical constituents. The Ca/Mg ratio in the study area ranged from 0.5 to 6.3; generally, a value of 1 represents dolomite dissolution, and a value higher than 1 suggests calcite as the dominant contributor. The plot showed the relation between Ca/Mg and Na/Cl and exhibits a greater variation (Fig. 7c). Most DW samples are found in the calcite and dolomite dissolution zone; the same is true for other sources such as HP, springs and SW. In the case of TW except for two samples, the rest show extensive silicate weathering (Fig. 7d). As is evident the Cai + Mgi versus total cations plot clearly displays all of the samples that are plotted in the Total Cations field. It’s possible that ion exchange processes have an impact on the various patterns that other samples exhibit. Ion exchange processes could be assessed using bivariate plots for (Cai + Mgi) vs. (HCO_3_ + SO_4_), (Na-Cl) vs. (Cai + Mgi) +(HCO_3_ + SO_4_) and Na/Cl and Cai + Mgi (Fig. 7e, f, and g). The majority of the samples, according to the results, fall into the HCO_3_ + SO_4_ field, which represents typical ion exchange processes (Fig. 7e), apart from a few samples that lay on 1:1 aquiline the rest deviate representing the dominance of silicate weathering. The positive trend indicated in Fig. 7f, and the negative correlation indicated by Fig. 7g also points towards the assumption that silicate weathering is the dominant factor at play in influencing the hydrochemistry in the study area^51,16^. The Piper-Hill diagram was utilized to analyze the hydrogeochemical evolution of the water within the research region^28^. Thus, the Ca-Mg-HCO_3_ type hydro-chemical facies predominate in the study region, trailed by the Na-HCO_3_ facies (Fig. 7). Only one spring water sample is shown in the Ca-SO_4_ zone, while three TW and one spring water sample show Na-Cl type facies.

### Water quality index (WQI)

**Fig. 8:**
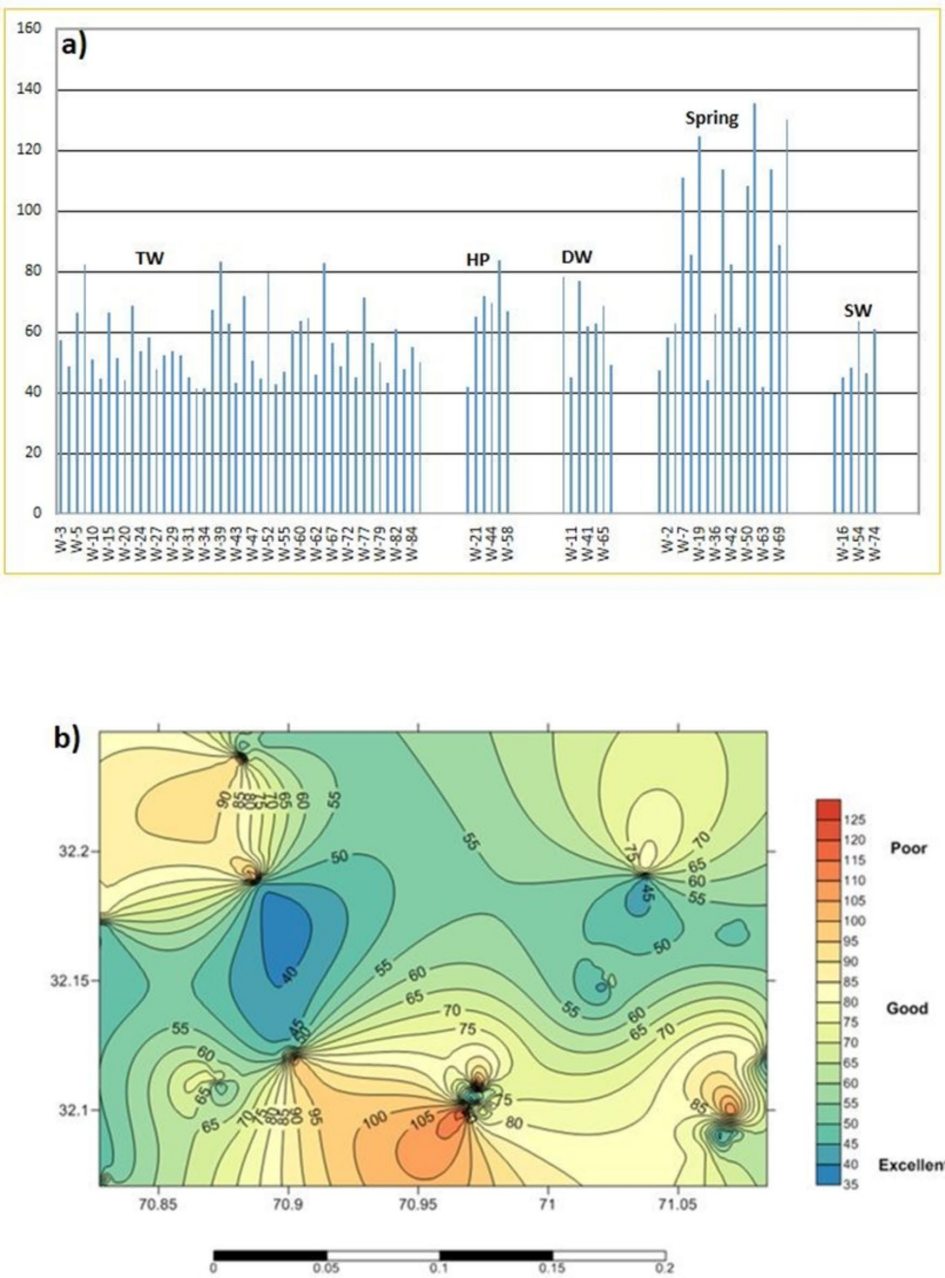
(**a**) the comparison of WQI values among sources and sampling sites and (**b**) the spatial distribution of WQI in the research area.

The WQI was conducted to evaluate the drinking water quality in the research area. According to the WQI value, water is categorized as excellent (0–295), good (215–510), moderate (520–725), poor (725–1600), and very poor (more than 1100) for domestic use and drinking^52^. The WQI was computed for each source to evaluate the general impact of chemical constituents and determine the water’s appropriateness for drinking. WQI assists in describing the combined effect of drinking water quality variables into a single figure that is further divided into quality classes described earlier^31^. The WQI in the research region varied from 17.20 to 132.76, with a mean of 52.36, i.e. ranging from excellent to poor water quality. WQI values of the TW, HP, DW, and spring ranged from 39 to 82, 43–83, 44–79, band 40–136, respectively (Fig. 8a and b). The spatial distribution of WQI in the research area is presented in Supporting Information Fig. 8b. As evident from the water sources in the south of the research region showed poor water quality, the majority of the region has good water quality, while the excellent waters are confined to a smaller fraction, mostly around Gohal. Overall, the research region’s samples have been categorized as excellent and good water, with the exception of 5 spring water samples. It suggests that in certain places, water must be treated before consumption and that the study area’s water quality may be impacted by roadside salt, rock weathering, rock-water interactions, and reverse ion exchange processes^53^. Furthermore, the variation in water quality highlights the importance of consistent monitoring and assessment to ensure public health safety^43^.

### Multivariate statistical analysis

**Fig. 9:**
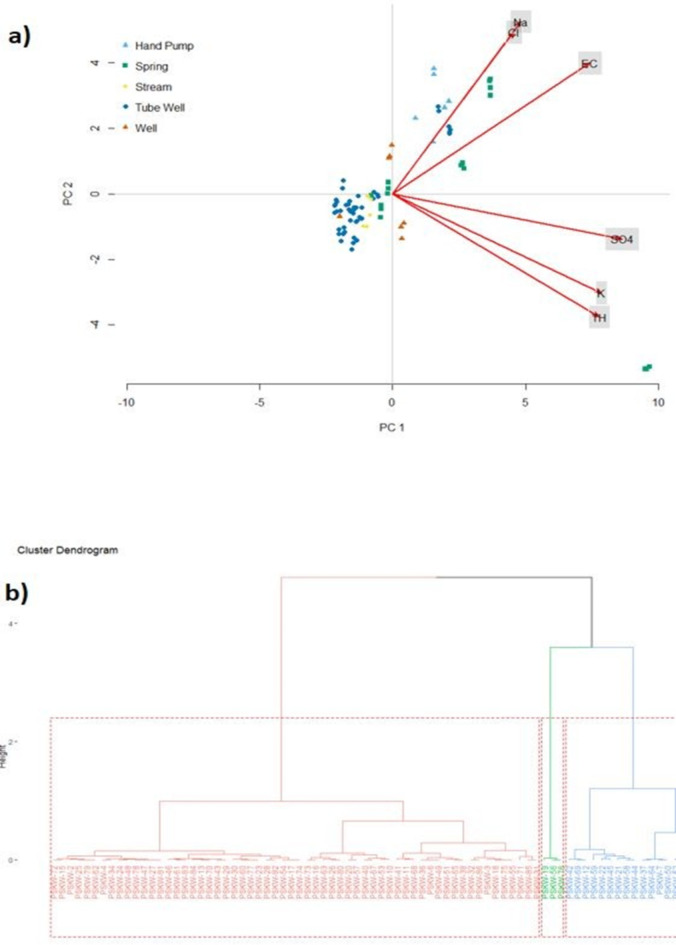
(**a**) PCA biplot showing the distribution of sampling sites and parameters on the first two components, (**b**) Cluster dendrogram showing the distribution of sampling points based on attributes.

The Pearson coefficient matrix is displayed for assessing the correlation among the chemical constituents in the water of the study region. The matrix is depicted in Table 2, which highlights the existence of noteworthy correlations between the cations and anions within the water. As is shown significant correlation pairs for TDS-HCO_3_, TDS-SO_4_, TDS-Cl, and TDS-Na were observed. Similarly, Ca shows a very strong correlation with SO_4_ and Mg. Other significant pairs are K-Ca, K-Mg, Na-Cl, and K-SO_4_. The principal component analysis was also carried out on the data, and Keyxser-Meyer Olkin (KMgD)^54^ and Bartlett’s sphericity index test^55^ were used to assess the data’s suitability for PCA analysis. The PCA analysis’s results are displayed as a biplot of indicators and variables in Fig. 9a, as is evident Na, Cl, EC, SO_4_, K, and TH have a major influence on PC1 indicating geogenic sources. The PC scores of indicators (sampling sites) were shown on the biplot based on their sources. The biplot divided the sampling sites into groups based on chemical attributes. All of the HP and the majority of the spring water samples show an influence on PC1. A cluster dendrogram based on the eigenvalues of PCA analysis was constructed to identify the cluster of sampling sites with similar hydro-chemical characteristics (Fig. 9b). Samples from all the sources were grouped together in Cluster 1, which was predominately made up of TW samples. Cluster 2 is made up exclusively of spring water samples; the three samples in this cluster do have greater WQI values and anomalously higher concentrations. Additionally, cluster 3 consists essentially of 19 samples derived from spring water and HP.

**Table 2:** Pearson correlation matrix of physicochemical parameters in water of the study area.

Parameters	pH	Eh	TDS	HCO_3_	SO_4_	Cl	Ca	Mg	Na	K	SIO_2_	Cr	Mo	Ni	Cu	*P*	Zn	Fe
pH	1																	
Eh	− 0.290	1																
TDS	0.11	0.003	1															
HCO3	0.15	0.017	0.346	1														
SO4	-0.08	0.002	0.575	-0.183	1													
Cl	-0.13	-0.064	0.559	-0.029	0.548	1												
Ca	-0.48	0.172	0.116	-0.241	0.534	0.079	1											
Mg	-0.46	0.2	0.203	-0.259	0.566	0.286	0.798	1										
Na	0.03	-0.08	0.419	0.212	0.326	0.688	-0.075	-0.019	1									
K	-0.32	0.2	0.23	-0.19	0.448	0.354	0.476	0.555	0.38	1								
SIO2	-0.145	-0.32	-0.209	-0.083	-0.098	-0.015	-0.09	0.054	-0.008	-0.091	1							
Cr	0.04	0.15	0.034	-0.027	0.143	-0.031	0.082	0.098	0.114	0.146	-0.005	1						
Mo	-0.06	-0.13	-0.11	-0.027	-0.011	-0.101	-0.03	-0.019	-0.114	0.03	0.01	0.05	1					
Ni	0.2	0.03	0.024	0.081	0.018	0.096	-0.097	-0.15	0.044	-0.142	-0.062	0.067	-0.116	1				
Cu	0.21	-0.03	-0.037	-0.005	0.065	-0.042	-0.006	0.006	0.048	-0.07	-0.058	0.024	-0.005	0.044	1			
P	-0.07	0.13	-0.188	0.083	-0.208	-0.214	-0.021	-0.128	-0.16	-0.074	-0.121	-0.078	-0.033	0.004	0.142	1		
Zn	0.11	-0.07	-0.001	-0.116	0.073	0.078	-0.091	0.012	0.146	0.066	-0.02	0.308	-0.032	-0.146	0.037	0.029	1	
Fe	0.15	-0.045	-0.127	0.151	-0.219	-0.131	-0.137	-0.291	0.097	-0.317	0.268	-0.029	-0.078	0.005	-0.063	-0.048	-0.074	1

#### Health risk assessment

**Fig. 10:**
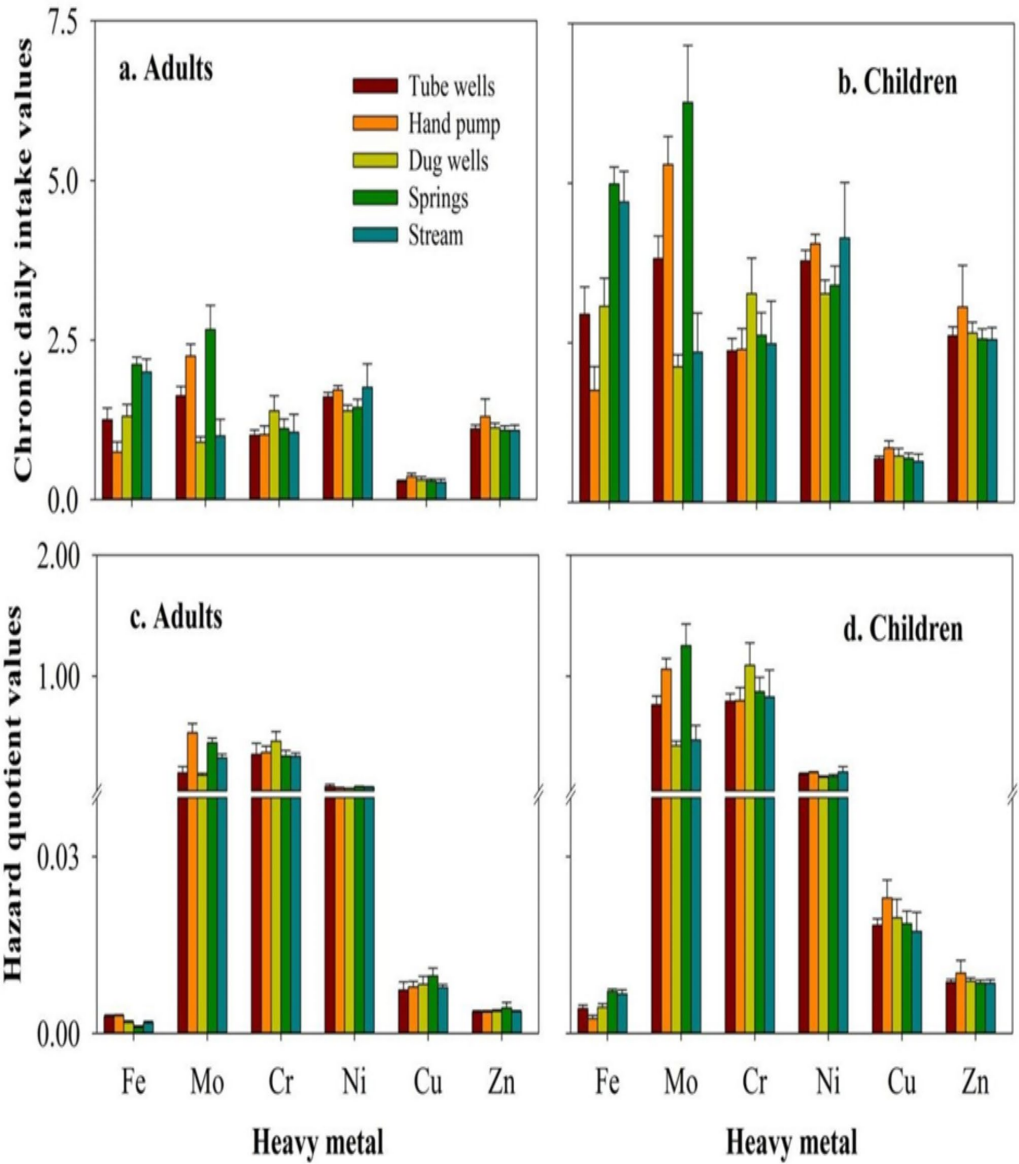
Risk assessment indices (**a**) chronic daily intake values (µg/kg-day) for adults, (**b**) chronic daily intake values (µg/kg-day) for children, (**c**) hazard quotient values for adults, and (**d**) hazard quotient values for children.

Health risk assessments for both adults and kids were computed using the CDI and HQ, and findings were compiled in Fig. 10a–d. The findings revealed that, when compared to other sources, children’s intake of Cr was greatest when they drank water from the spring, while adults’ intake of Cu was lowest when they drank water from TW. (Fig. 10 a and b). Greater CDI levels of Cr could be ascribed to its high contamination in spring water. Because of their smaller bodies, children’s intake was higher. Children’s highest CDI levels were found to be consistent by water ingestion with the previous research work by^2^. The findings showed the highest HQ levels of Mo, succeeded by Cr for children through water ingesting from the spring and DW, while the lowest of Fe for adults through TW when matched to other causes (Fig. 10 c and d). The Mo and Cr exceeded the children’s acceptable limit for spring and DW water ingestion only, with HQ > 1. Several factors were identified as contributing to the HQ values, including intake, metal toxicity, RfD levels, and personal sensitivity^56^.

### Water quality for irrigation purposes

**Fig. 11:**
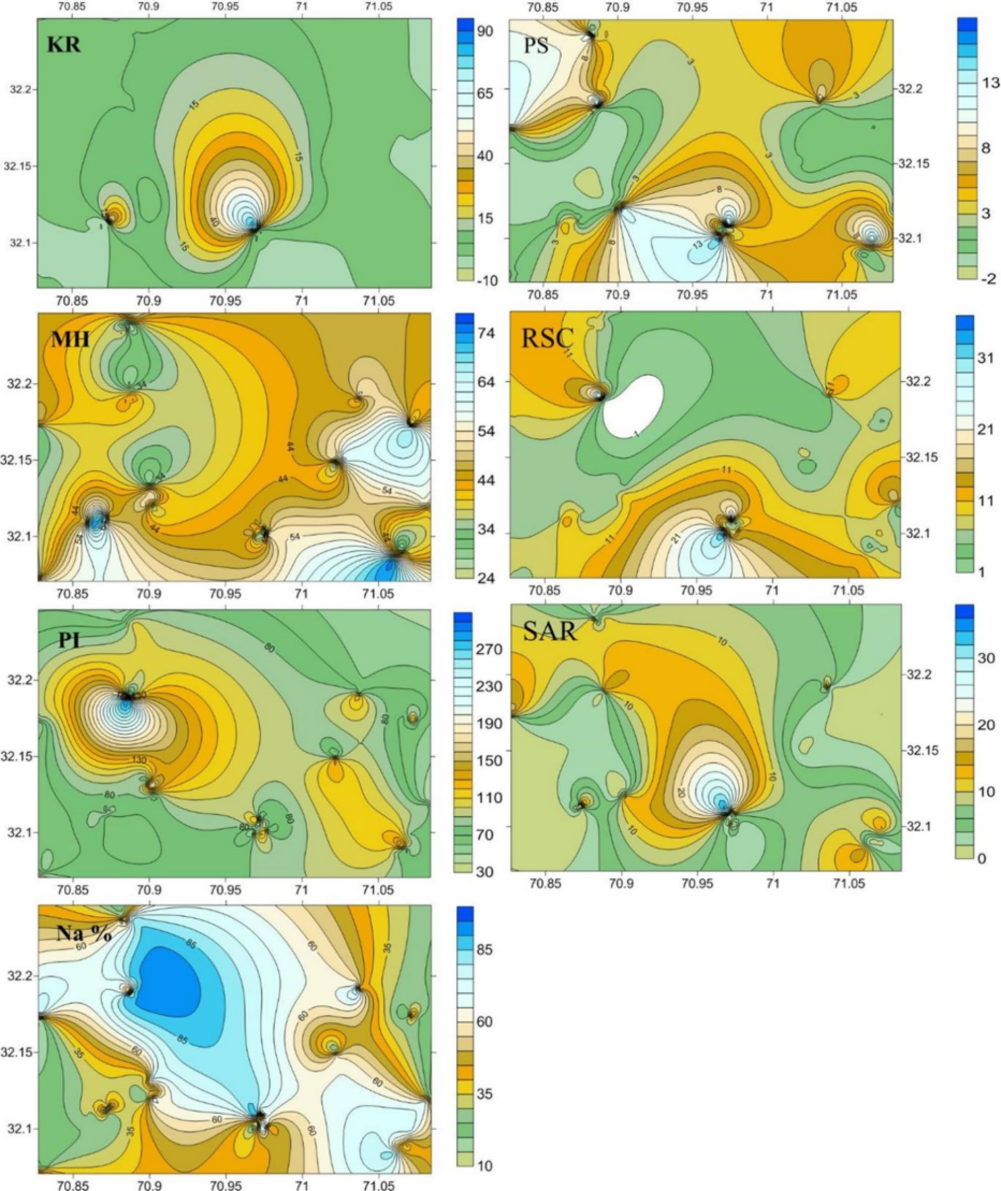
Spatial distribution of irrigation water quality parameters along with sampling sites of the study area.

Surface and subterranean water is typically the primary source of agricultural use in semi-arid regions. Thus, it is essential to comprehend the appropriateness of water for irrigation purposes^43^. In this case, the appropriate irrigation parameters in the present research are MH, SAR, RSC, %Na, PS, PI, RBSC, and KR. The geographic arrangement of irrigation water quality variables discussed above is presented in Fig. 11^57^. has established a permeability index (PI) for evaluating whether water is acceptable for irrigation, one of the key indices to evaluate the appropriateness of water for irrigation. The mean PI level in the research region was 58.76, and the values varied from 26.84 to 81.11. As per Fig. 11 and the Doneen classification, over 99% of the research region is categorized as excellent. KR is also a crucial criterion for irrigation suitability. The range of the KR values in the research area was 0.07 to 3.23, with a median score of 0.92. About 84.5% of the study region was found to be < 1, showing that groundwater is a good class for irrigation suitability based on^58^ classification (Fig. 11). The remaining study area is divided into two categories: appropriate and inappropriate. All water samples in the study region showed a KR value of > 1, with HP water having the highest mean KR value compared to other sources^59^. asserts that an excessive RSC concentration in water causes the soil to become more alkaline, rapidly turning the impacted areas into barren land. Within the study area, RSC values varied from − 26.653 to 51.501 meaq/L, with an average of -56.762 mbeq/L that indicates that all samples fall into the good category of RSC (< 11.25 RSC level). According to^22^, a negative RSC value indicates that Ca and Mg have a significant influence in the research area. Consequently, when evaluating the quality of groundwater, the concentrations of Mg and Ca are also crucial. Compared to Ca, Mg is more detrimental to soil. That’s why it’s essential to calculate the MH to decide whether the water is suitable for irrigation. Two classes, which include Excellent and Good, are shown on the MH map in Fig. 11. The excellent class (less than 50) includes nearly 86% of the research area; the remaining portion falls into the good category of MHR. The TW and HP water pose a considerable Mg hazard to the soil as compared to other sources. Na% is a key statistic that helps in ascertaining the risks associated with high Na concentrations in irrigation water. A high concentration of Na in water can have a significant effect on the soil’s aeration, infiltration, and structure. In general, Na% values less than 20 are generally regarded as excellent, 20–40 as good, 40–60 as acceptable, 60–80 as dubious and a value greater than 80 as inappropriate. The majority of the water sources were observed in good and excellent while few were in the permissible category. Moreover, SAR sheds light on the risks associated with high Na levels in irrigation water^60^. A SAR level of more than 226 is regarded as inappropriate for irrigation; the excessive levels of SAR in the water of the research region may impact the permeability of the soils and reduce the uptake of water by plants. The results for RSC are similar to those of SAR, in that all the samples in the research region have RSC levels greater than the permissible threshold (> 2.55 maeq/L) and indicate the unsuitability of the water^61^. Excessive RSC can cause NaHco_3_ to settle in the soil and reduce its fertility. SAR, Na%, and RSC show a high level of risk owing to the higher amount of Na in the study area’s water. The PS Index ascertains the dominance of either SO_4_ or Cl ions in the water. A value of PS > 3 meq/L is considered unsafe^57,62^. All the samples in the HP source pose concerns for their suitability as an irrigation water source, while samples (76%) of the TW do not pose any hazard based on PS values.

## Conclusion

Surface and subterranean water quality is a key factor in assessing human development and well-being, as these sources are critical for household, agricultural, and industrial purposes worldwide. The degradation of water quality, whether due to natural geological processes or human-induced activities, poses serious challenges to public health, agriculture, and ecosystem sustainability. This study focused on evaluating the hydrochemistry of surface and groundwater in the arid and semi-arid regions of the Talagang District, providing valuable insights into water quality and its suitability for various uses.

The water in the study area was found to be of near-neutral, low-metal type, primarily governed by rock weathering processes, with Ca-Mg-HCO₃ facies predominating, followed by Na-HCO₃ facies. The hydrochemical characterization revealed that ion exchange, rock-water interaction, and silicate weathering are significant factors influencing water composition. Seven irrigation water quality indices were utilized to assess the suitability of water for agricultural purposes. Sodium-based indices, including Kelly’s Ratio (KR), Sodium Percentage (Na%), Residual Sodium Carbonate (RSC), and Sodium Adsorption Ratio (SAR), exceeded permissible thresholds in all samples, highlighting elevated sodium levels that pose risks to soil structure and crop productivity. On the basis of Potential Salinity (PS), all hand pump (HP) samples were deemed unsuitable for irrigation, while Permeability Index (PI) values indicated that most samples were either safe or marginally suitable for irrigation.

The Water Quality Index (WQI) values categorized most samples as belonging to the “good” water quality class, with a smaller proportion classified as “excellent” or “poor.” These findings suggest that water in the study area is generally suitable for drinking, with no significant restrictions recommended. However, proper treatment is necessary for irrigation purposes to mitigate sodium hazards, ensure soil fertility, and sustain agricultural output. Additionally, the Human Health Risk Assessment (HHRA) findings indicated that children are at greater risk from heavy metal exposure compared to adults, emphasizing the need for targeted interventions to reduce health risks.

This research not only provides a detailed evaluation of the current water quality in the Talagang District but also offers critical guidance for policymakers, water management authorities, and local stakeholders. It underscores the importance of integrated approaches to water quality monitoring, management, and remediation. Specific recommendations include constructing fully covered septic tanks, promoting the use of natural fertilizers instead of synthetic ones, and implementing effective sewage drainage systems to minimize contamination risks. Furthermore, this study contributes to a deeper understanding of the complex hydrochemical dynamics in semi-arid regions. By combining Water Quality Index (WQI) and Human Health Risk Assessment (HHRA) tools, it presents a robust framework for assessing water quality and its implications for public health, agriculture, and environmental sustainability. These findings serve as a foundation for future research and policy development, emphasizing the need for sustainable water management practices to support human and ecological well-being in the region and beyond.

## Data Availability

The datasets used and/or analysed during the current study available from the corresponding author on reasonable request.
